# Effects of topiramate on neural responses to alcohol cues in treatment-seeking individuals with alcohol use disorder: preliminary findings from a randomized, placebo-controlled trial

**DOI:** 10.1038/s41386-021-00968-w

**Published:** 2021-02-08

**Authors:** Reagan R. Wetherill, Nathaniel Spilka, Kanchana Jagannathan, Paige Morris, Danielle Romer, Timothy Pond, Kevin G. Lynch, Teresa R. Franklin, Henry R. Kranzler

**Affiliations:** 1 Department of Psychiatry, Perelman School of Medicine, University of Pennsylvania, Philadelphia, PA USA; 2 Mental Illness Research, Education and Clinical Center, Veterans Integrated Service Network 4, Crescenz VAMC, Philadelphia, PA 19104 USA

**Keywords:** Translational research, Predictive markers

## Abstract

Topiramate, a GABA/glutamate modulator, is efficacious in reducing alcohol consumption, though the mechanisms underlying this effect are not well characterized. This study analyzed functional magnetic resonance imaging (fMRI) data from 22 heavy drinkers enrolled in a 12-week placebo-controlled, randomized clinical trial of topiramate to examine the effects of topiramate on alcohol cue-elicited brain responses, craving, and heavy drinking in individuals with DSM-5 alcohol use disorder. Patients were randomized to receive either topiramate (maximal daily dosage of 200 mg/day) or placebo and were administered an fMRI alcohol cue-reactivity task at baseline (before starting medication) and after 6 weeks of double-blind treatment. Analyses compared the topiramate (*n* = 12) and placebo (*n* = 8) groups on (1) the change in brain responses during alcohol cue exposure (vs non-alcohol cues) within five a priori regions of interest related to reward—the bilateral and medial orbitofrontal cortex (OFC) and bilateral ventral striatum (VS) and (2) change in craving and heavy drinking days (HDDs) from baseline and scan 2. Topiramate, relative to placebo, reduced alcohol cue-elicited activation of the left VS, bilateral OFC, and medial OFC, alcohol cue-elicited craving, and HDDs between baseline and 6 weeks of treatment. The reduction in alcohol cue-elicited activation in the medial OFC correlated with reductions in craving, and reduced activation in the right VS, right OFC, and medial OFC correlated with the reduction in HDD. This preliminary study provides evidence that topiramate’s attenuation of alcohol cue-elicited brain activation and craving are key elements of the drug’s neurobiological mechanism of action in reducing heavy drinking.

## Introduction

Alcohol misuse and alcohol use disorder (AUD) are leading risk factors for premature death and disability [1]. In the United States, an estimated 16.5 million adults engage in heavy alcohol use (consuming 5 or more drinks for males or 4 or more drinks for females on 5 or more days during the preceding month), and 14.4 million meet criteria for an AUD [2]. Although there are effective treatments for heavy drinking and AUD, the majority of affected individuals receive no treatment, and among those that do, up to 70% return to drinking within the first year following treatment [3].

Exposure to alcohol-related cues contributes to continued alcohol consumption and relapse [4–6]. An extensive neuroimaging literature has examined brain responses to alcohol cues in individuals at risk for or diagnosed with AUD [5, 7–10]. According to a quantitative meta-analysis and systematic review, AUD is associated with robust alcohol cue-induced activation in reward-related mesolimbic and prefrontal brain regions, including the ventral striatum (VS) and orbitofrontal cortex (OFC), and cue-elicited activation of the VS is most frequently correlated with behavioral measures and reduced with treatment [6].

One promising medication being studied as a treatment for AUD is topiramate [11], which in several clinical trials reduced heavy drinking in individuals with problem drinking or AUD [12–16]. We previously reported that topiramate reduced heavy drinking among individuals who sought to reduce their drinking, with the effect moderated by a single nucleotide polymorphism (SNP; rs2832407) in *GRIK1*, encoding the kainate GluK1 receptor subunit [16]. In a follow-up replication study, we attempted to validate the pharmacogenetic findings and the efficacy of the 200 mg/day dosage of topiramate for treating AUD [17]. Specifically, we conducted a prospective randomized pharmacogenic study of topiramate, in which patients were randomized to receive topiramate or placebo for 12 weeks based on their genotype. Although the moderating effect of rs2832407 genotype was not replicated, patients treated with topiramate showed a nearly twofold reduction in heavy drinking days (HDDs) compared to those who received placebo. Despite these compelling findings, little follow-up has been conducted to determine the neurobiological mechanisms underlying topiramate’s effect on heavy drinking.

Topiramate is an anticonvulsant medication with multiple pharmacologic effects that inhibit neuronal activity, including enhancing gamma-aminobutyric acid (GABA) activity, suppression of voltage-sensitive Na^+^ channels, and antagonism of glutamate transmission through effects at AMPA/kainate receptors [18, 19]. While the mechanisms underlying the therapeutic effects of topiramate in reducing heavy drinking remain unclear, it has been hypothesized that topiramate reduces heavy drinking by inhibiting alcohol-induced dopamine release in the reward-related mesolimbic dopamine pathways by enhancing GABA neurotransmission and/or inhibiting glutamatergic neurotransmission [20, 21]. This suppression of alcohol-induced dopamine release could decrease the reinforcing effects of alcohol. Over time, repeated reductions in alcohol-induced reinforcement may devalue the rewarding properties of alcohol cues and attenuate the motivation (i.e., craving) to consume alcohol [12].

Several studies have examined the effects of topiramate on alcohol craving. In randomized controlled trials [12, 14] and open-label studies [22, 23], topiramate reduced alcohol craving in treatment-seeking heavy drinkers compared to placebo. However, human laboratory and other studies report no effect of topiramate on general craving or alcohol cue-elicited craving [24] or a reduction in craving only while participants were consuming alcohol [25]. Inconsistencies in topiramate’s effects on craving may be related to differences in methodologies and/or the populations studied. Specifically, studies conducted in individuals with AUD have shown that topiramate blunts craving, while human laboratory studies in nontreatment-seeking heavy drinkers report little or no effect on craving. Given these inconsistent findings, a more objective, neurobiologically informative measure of topiramate’s effects on alcohol-related behaviors and measures may provide insight into the mechanism by which topiramate reduces heavy drinking.

The current study, which was part of a larger 12-week RCT [17], used an objective, neurobiological approach to compare the effect of topiramate and placebo on brain responses to alcohol cues, alcohol cue-elicited craving, and heavy drinking in treatment-seeking heavy drinkers with AUD. Patients were tested twice using *pseudo*-continuous arterial spin labeling (*p*CASL) perfusion functional magnetic resonance imaging (fMRI) during alcohol and non-alcohol cue exposure, before starting treatment and after 6 weeks of study medication. We hypothesized that topiramate, relative to placebo, would reduce alcohol cue-elicited activation of reward-related brain areas, alcohol cue-elicited craving, and heavy drinking and that these effects would be intercorrelated.

## Methods

### Overview

The current study was a substudy of a prospective, randomized, pharmacogenetic trial investigating the efficacy of topiramate in reducing the frequency of heavy drinking (ClinicalTrials.gov: NCT02371889; [17]). The University of Pennsylvania (UPenn) Institutional Review Board approved all procedures, and the study was carried out in accordance with the Declaration of Helsinki. All patients gave written informed consent before participating. Patients were randomly assigned equally to treatment groups by staff who were not involved in providing treatment, using a random allocation provided by a statistician. Double-blind treatment conditions were maintained throughout the study. The study included an additional informed consent and screening visit for the fMRI. The fMRI scans were conducted before starting medication (at the baseline visit) and 6 weeks later (i.e., after the study medication was increased to a maximal tolerated dosage for one full week).

### Patients

Treatment-seeking individuals who provided informed consent and were eligible for the larger clinical trial at the UPenn site (*N* = 164) were recruited to participate in the current study. Interested patients were screened for the following inclusion criteria: age 18–60; self-identified European ancestry; a weekly average of ≥24 standard drinks (men) or ≥18 standard drinks (women); a current DSM-5 diagnosis of AUD; a goal of either stopping drinking or reducing their drinking to safe levels, defined as no more than 3 standard drinks per day and 12 drinks per week for men and no more than 2 drinks per day and 8 drinks per week for women; ability to read English on at least an 8th-grade level; no gross evidence of cognitive impairment; intelligence quotient of ≥80, as estimated by the 2-subtest score of the Wechsler Abbreviated Scale of Intelligence (WASI) [26], and willingness to provide written, informed consent to participate and to name someone who could be contacted to locate the patient if he or she could not be reached. In addition, women of childbearing potential had to be non-lactating, practicing a reliable method of birth control, and provide a negative urine pregnancy test at screening and monthly during the study. Exclusion criteria included the presence of a current, clinically significant physical disease or abnormality on medical history, physical examination, or routine laboratory evaluation; a history of nephrolithiasis, glaucoma, or hypersensitivity to topiramate; treatment with carbonic anhydrase inhibitors or dolutegravir; a serious psychiatric illness or current treatment with a psychotropic medication or one aimed at reducing their drinking; a current diagnosis of drug (other than nicotine) dependence; a urine drug screen positive for recent use of opioids, cocaine, or amphetamines; a clinical presentation that indicated a need to be abstinent from alcohol (e.g., current gastritis, a recent or past history of severe alcohol withdrawal symptoms); history of head trauma or injury causing loss of consciousness lasting more than 5 min or associated with skull fracture or intracranial bleeding; history of seizure; history of stroke and/or stroke-related spasticity; the presence of magnetically active irremovable objects in the body; claustrophobia or other medical condition preventing the individual from lying in the MRI for approximately 1 h. From the larger study, 38 eligible patients agreed to take part in the brain imaging study and provided written, informed consent to participate. Of these 38 patients, 29 were randomly assigned to treatment with topiramate or placebo. Supplementary figure 1 is a CONSORT diagram, which shows the reasons that 18 potential patients were excluded from the study and analyses.

### Assessment, randomization and intervention

Inclusion and exclusion criteria were initially assessed by phone, after which prospective patients were invited to an in-person informed consent and clinical trial screening visit whose methods are described in detail elsewhere [17]. Briefly, potential patients completed psychological and behavioral assessments and underwent a medical and psychiatric history, physical examination, routine clinical laboratory testing, a urine drug screen, and, if appropriate, pregnancy testing. Assessments included a modified version of The Structured Clinical Interview for DSM-IV (SCID-IV [27]) that incorporated DSM-5 criteria for AUD [28]. The Timeline Follow-back Method (TLFB [29]) was used to estimate the number of drinks per drinking day, drinking days (DDs) and heavy DDs. The Short Index of Problems (SIP [30]) assessed alcohol-related problems. Patients eligible for the clinical trial were screened and recruited to participate in the current fMRI study. Interested patients were invited to a second screening visit for further assessment, where they provided informed consent for the fMRI study. During this session, a trained research technician administered the WASI [26] to estimate the patients’ intelligence quotient. Eligible patients were then scheduled for their baseline treatment appointment and first fMRI scan visit. They were instructed to abstain from alcohol the night before fMRI scan visits.

At the baseline/fMRI scan visits, patients completed questionnaires and met with the study physician or nurse practitioner. The study nurse administered the Clinical Institute Withdrawal Assessment for Alcohol-Revised (CIWA-Ar [31]), delivered the first medical management session, and dispensed study medication. Patients were instructed to start the study medication that evening, following the first fMRI scan. Patients received either topiramate (25 mg daily for 1 week with gradual weekly increases to 100 mg twice daily during weeks 6–12 followed by a gradual 1-week taper to zero) or placebo. Study medications were identically encapsulated and dispensed in pill bottles. Patients and investigators were blind to medication assignment. After randomization and the first fMRI scan, patients returned weekly for the first 6 weeks then biweekly for 6 weeks. At each visit, patients received medical management [32], a brief psychosocial intervention aimed to enhance treatment adherence.

### Alcohol cue-reactivity task

Patients were scanned at baseline (immediately before ingesting the first medication dose) and after at least 6 weeks of medication (mean = 6.65 weeks, standard deviation (SD) = 1.04). At each scan session, patients were breathalyzed to ensure that they had not recently consumed alcohol and were administered the CIWA-Ar to assess withdrawal severity. No patient had a breath alcohol content >0 or a CIWA-Ar score >3. Patients were then escorted to the fMRI scanner where they completed an fMRI safety form and were positioned in the scanner. *p*CASL perfusion fMRI, a quantitative estimate of cerebral blood flow (CBF) and indirect measurement of neural activity [33], assessed brain activation in response to the alcohol cue exposure. During each scan session, participants completed a resting baseline scan, a 10-min non-alcohol cue *p*CASL scan, a 10-min alcohol cue *p*CASL scan, and a high-resolution structural scan. Subjective ratings of alcohol craving were assessed before and after each cue video.

The alcohol cue video included individuals of different races, ages, and sex consuming alcohol and using explicit language designed to induce an appetitive desire for an alcoholic beverage. The non-alcohol cue video was similar in content, except that it did not portray alcohol consumption or other alcohol-related stimuli. The non-alcohol cue video was always shown before the alcohol cue video to minimize interference due to ‘carryover’ arousal initiated when alcohol cues are shown first, which can affect responses to non-alcohol cues [34, 35].

### Imaging data acquisition and preprocessing

Imaging data were acquired on a 3.0 Tesla whole-body scanner (Siemens AG, Erlangen, Germany). For co-registration of the functional data, a T1-weighted three-dimensional (3D) high-resolution MPRAGE scan was acquired with field of view (FOV) = 250 mm, TR/TE = 1620/3 ms, 192 × 256 matrix, slice thickness 1 mm. *p*CASL perfusion fMRI sequence was used for alcohol cue and non-alcohol cue data acquisition. Interleaved images with and without labeling were obtained with a labeling time = 1.5 s, post labeling delay = 1 s, FOV = 22 cm, matrix = 64 × 64 × 31, TR/TE = 4110/20 ms, flip angle = 90°, slice thickness = 4.8 mm and voxel size = 3.4 × 3.4 × 4.8.

Data preprocessing was carried out using SPM-based arterial spin labeling (ASL) data processing toolbox [36] run under a MATLAB R2020 environment. Each participant’s ASL image pairs were realigned to correct for head motion, and the mean image was co-registered to the high-resolution structural image using the FMRIB Software Library (FSL) Boundary Based Registration (BBR) approach [37]. A binary mask comprising GM, WM, and CSF was applied to limit the CBF computation within the brain voxels. For both alcohol cue and non-alcohol cue stimuli, 100 CBF image series were generated from the 100 label/control ASL image pairs by pairwise control-label subtraction using a single compartment model with recommended parameters [38]. Subsequently, the structural image was spatially normalized to the Montreal Neurological Institute (MNI) standard brain using FSL non-linear registration, and the resulting BBR transformation was used to align CBF images to MNI space. The normalized images were spatially smoothed with a 5 mm full width half maximum Gaussian kernel. In addition, each subject was examined for quality evaluation index (QEI) [39], with output values between 0 (no signal) and 1 (highest signal). The images with QEI of 0.7 or above were included in all our analyses.

### Regions of interest (ROIs)

**Fig. 1:**
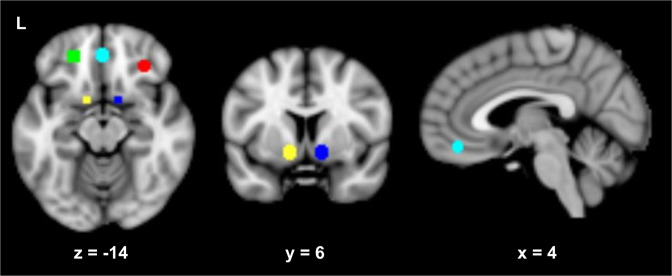
Representation of regions of interest (ROIs). Regions of interest: left (green), medial (light blue), and right (red) orbitofrontal cortex; left (yellow) and right (blue) ventral striatum.

Based on previous studies of alcohol cue reactivity [6, 40, 41], ROIs were defined a priori as 6-mm radius spheres centered at [12 6 −9] (right VS); [−12 6 −9] (left VS); [32 34 −11] (right OFC); [−22 42 −12] (left OFC); and [0 43 –16] (medial OFC) in MNI space (Fig. 1).

### Statistical analysis

Continuous demographic variables were summarized by calculating means and standard error measurements. Independent samples *t* tests compared topiramate and placebo groups on continuous variables. Nominal demographic variables were summarized by calculating proportions and compared across groups using chi-square tests.

Contrasts between alcohol cue versus non-alcohol cue sets were defined in the general linear model to assess the voxel-by-voxel CBF difference for each patient. Using the corresponding parametric maps of this contrast, random effects analysis was employed to test the main effect of condition (alcohol cue vs non-alcohol cue) in topiramate and placebo groups with a statistical parametric map of the *t*-statistic at each voxel for population inference for each session within the five ROIs.

For each patient and each imaging scan session, average quantitative CBF values (ml of blood/100 g of tissue/min) of the alcohol versus non-alcohol contrast (i.e., neural responses to alcohol cues) were extracted from the five a priori ROIs. The change in neural responses to alcohol cues from baseline to scan 2 for each ROI was computed, and the change in neural response to alcohol cues in topiramate and placebo groups was compared using the Wilcoxon rank sum test (*p* < 0.05). Similarly, analyses were conducted for change in alcohol cue-induced craving from baseline to scan 2 and change in HDD (≥5 standard drinks in one day for men, or ≥4 standard drinks in one day for women) during the week prior to each scan session. All participants included in the analysis provided drinking data for the week prior to scan 2. HDD was chosen as the drinking outcome because topiramate reduced %HDD in our two clinical trials of topiramate for reducing heavy drinking [16] and treating AUD [17]. Correlations between the change in average quantitative CBF values and the change in HDD and in alcohol cue-induced craving were assessed for the full sample and each treatment group separately using the Spearman’s rank correlation coefficient *ρ*, and ordinary nonparametric bootstrap analysis was used to derive the 95% confidence interval (CI) values.

## Results

### Sample characteristics

Demographic and baseline characteristics of the two groups are presented in Table 1. The topiramate and placebo patients did not differ significantly on demographic variables or drinking during the month before the first scan (baseline).

**Table 1:** Demographic and baseline data.

	Topiramate	Placebo	Test for difference
*N* (M/F)	8/4	6/2	*χ*^2^(1,19) = 0.16, *p* = 0.69
Age, yr	50.5 (8.1)	45.1 (13.2)	*t*(18) = −1.14, *p* = 0.27
Education, yr	17.3 (1.6)	16.8 (1.7)	*t*(18) = 0.54, *p* = 0.60
Drinking days (past month)	24.3 (5.1)	27.0 (3.5)	*t*(18) = 1.32, *p* = 0.20
Drinks per drinking day (past month)	7.0 (3.3)	5.8 (1.6)	*t*(18) = −0.57, *p* = 0.57
Heavy drinking days (past month)	19.9 (7.7)	17.9 (8.0)	*t*(18) = −0.91, *p* = 0.37
SIP Score (90 day before screening)	12.0 (6.4)	14.5 (11.9)	*t*(18) = 0.62, *p* = 0.55

One patient each from the topiramate and placebo groups endorsed smoking 2–3 cigarettes per day.

*SIP* Short Index of Problems.

### Medication adherence and maximal dosage achieved

Adherence rates between baseline and scan 2 were high in both medication groups: the placebo group averaged 7.0 days of medication ingestion per week, while the topiramate group averaged 6.8 days (standard deviation (SD) = 0.6). During the week prior to scan 2, eight topiramate patients (66.7%) and seven placebo patients (87.5%) reached the maximal dosage (200 mg) or the equivalent number of placebo capsules [*χ*^2^_(1)_ = 1.11, *p* = 0.60]. For patients who did not reach a dosage of 200 mg/day, the maximum tolerated dosage was used in the analyses.

### Primary outcomes

**Fig. 2:**
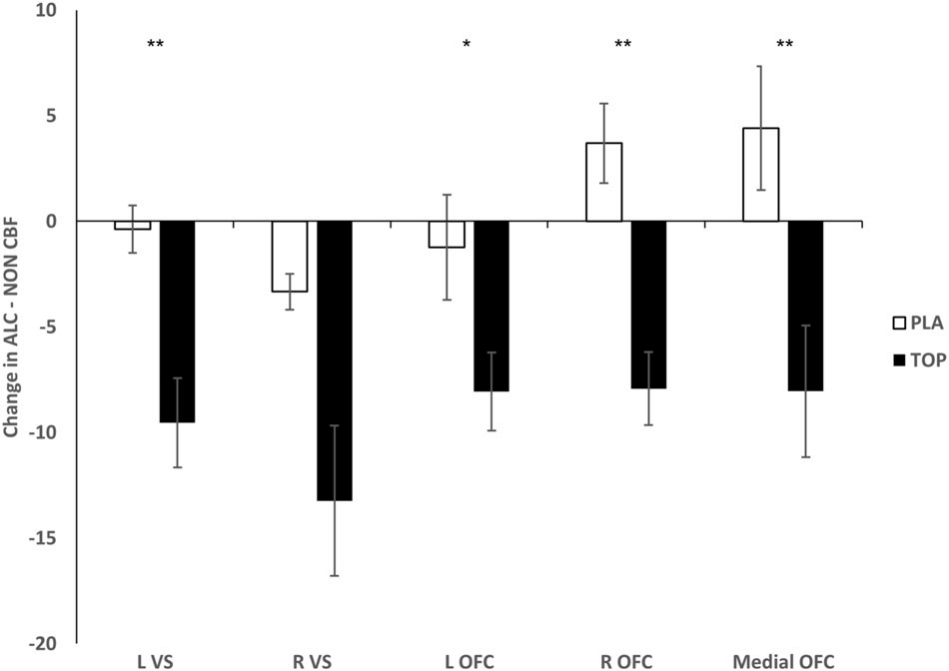
Change in alcohol cue-elicited activation (ALC-NON) from baseline to scan 2 in each medication group. Figures are mean ALC-NON cerebral blood flow values (±standard error). Topiramate, relative to placebo, reduced ALC-NON activation in the left ventral striatum (VS), bilateral orbitofrontal cortex (OFC), and medial OFC, but the reduction in the right VS did not reach significance. **p < 0.005; *p < 0.05.

**Fig. 3:**
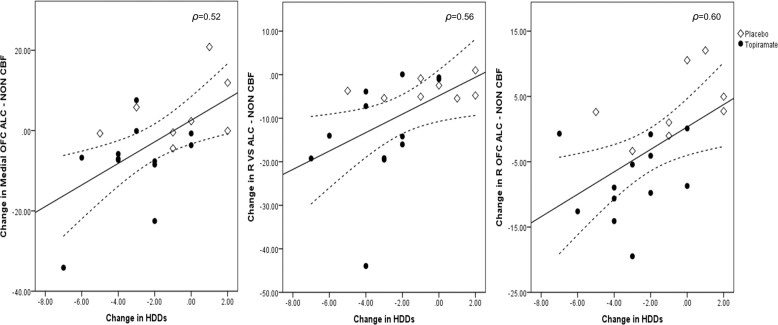
Scatter plot of change in alcohol cue-elicited activation (ALC-NON) from baseline to scan 2 in each medication group for the medial orbitofrontal cortex (OFC) (left), right ventral striatum (VS) (middle), and right OFC (right) with change in heavy drinking days (HDDs) from baseline to scan 2. Fit lines, 95% confidence intervals, and spearman correlations (*ρ*) are included for each region of interest.

Compared to placebo, topiramate significantly reduced neural responses to alcohol cues in the left VS (*z* = −2.93, *p* = 0.002), left OFC (*z* = −2.01, *p* < 0.05), right OFC (*z* = −3.24, *p* < 0.001), and medial OFC (*z* = −2.82, *p* = 0.003) (Fig. 2). The change in the right VS did not reach significance (*z* = −1.85, *p* = 0.06). Similarly, individuals treated with topiramate, relative to placebo, reported reduced alcohol cue-elicited craving (*z* = −2.00, *p* = 0.05) and reduced HDDs from baseline to the second scan (*z* = −2.10, *p* = 0.04). The change in neural responses to alcohol cues correlated with the change in alcohol cue-elicited craving in the medial OFC (*ρ* = 0.60, 95% CI [0.14, 0.88], *p* = 0.005) and the change in HDD in the right VS (*ρ* = 0.56, 95% CI [0.30, 0.77], *p* = 0.01), right OFC (*ρ* = 0.60, 95% CI [0.21, 0.83], *p* = 0.006), and medial OFC (*ρ* = 0.52, 95% CI [0.12, 0.81], *p* = 0.02) (Fig. 3). Among topiramate-treated patients, the change in neural responses to alcohol cues correlated with the change in alcohol cue-elicited craving in the the medial OFC (*ρ* = 0.53, 95% CI [0.05, 0.81], *p* = 0.04) and the change in HDD in the right VS (*ρ* = 0.50, 95% CI [0.11, 0.84], *p* < 0.05). There were no significant correlations found for the placebo group. Given that age and baseline drinking could influence results, we conducted Quade’s rank analysis of covariance with age and baseline drinking as covariates. Findings did not change.

## Discussion

The goals of this preliminary study were to evaluate the effects of topiramate on brain responses to alcohol cues and their relation to alcohol cue-elicited craving and heavy drinking in treatment-seeking heavy drinkers with AUD. Consistent with previous reports and the larger randomized controlled clinical trial, topiramate reduced heavy drinking [12, 16, 17, 23]. Topiramate also attenuated alcohol cue-elicited craving and neural responses within dopaminergic mesocorticolimbic reward-related ROIs (i.e., left VS, bilateral OFC, and medial OFC) more than placebo. The change in neural response to alcohol cues in the medial OFC was associated with the change in alcohol cue-elicited craving, and the change in neural responses to alcohol cues in the right VS, right OFC, and medial OFC was associated with the change in heavy drinking days. Among topiramate-treated patients, the change in neural response to alcohol cues in the medial OFC was associated with the change in alcohol cue-elicited craving, and the change in neural responses to alcohol cues in the right VS was associated with the change in heavy drinking days.

The VS and OFC are key brain regions mediating cue reactivity. Preclinical research has shown that phasic firing of dopaminergic neurons projecting from the ventral tegmental area to the VS is critical for behavioral conditioning [42], and ventral striatal activation is associated with reward anticipation when salient cues are present [43, 44]. The OFC receives direct and indirect projections from the VS and other limbic regions involved with drug reinforcement [45, 46] and plays a prominent role in integrating sensory inputs and guiding motivated behavior [47]. Indeed, single-cell activity tracking of orbitofrontal output neurons indicates that neuronal activity of the OFC represents a long-term cue-reward associative memory that supports behavioral adaptation [48]. Thus, our findings showing that topiramate attenuated reduced alcohol cue-elicited brain responses in VS and OFC and the associated reduction in alcohol cue-elicited craving suggest that by weakening the association between alcohol cues and alcohol reward, topiramate reduced the salience of alcohol cues and motivation to consume alcohol.

Consistent with previous studies and the larger clinical trial, topiramate significantly reduced HDDs [16, 17], and these effects were apparent after the gradual 6-week increase to the maximal dosage. In a follow-up analysis, the effect of topiramate on HDDs persisted through the end of treatment (*z* = −2.30, *p* = 0.02). The weekly reduction in the topiramate group was 2–3 HDDs, which is clinically significant, as the frequency of heavy drinking is associated with alcohol-related negative consequences [49–51].

The reduction in HDDs correlated with the attenuation of brain responses to alcohol cues in the VS and OFC. Because our data are correlational, it is not possible to determine whether the attenuation of brain responses to alcohol cues in the VS and OFC is a cause or effect of the changes in heavy drinking. However, *post hoc* analyses examining whether changes in heavy drinking days were associated with changes in global resting CBF from baseline to scan 2 showed no correlation (*p* = 0.18). Although replication of these findings is needed, it is possible that topiramate reduces heavy drinking by reducing the value of alcohol cues and alcohol reward.

This study had several important strengths and limitations. It is the first neuroimaging study to examine the effects of topiramate in AUD. The topiramate and placebo groups were well matched. As a preliminary study, the sample size was small, and we did not correct for multiple ROIs. Efforts to replicate these findings in larger samples are warranted. The inclusion of a baseline scan and the within-subject design are strengths of the study. The baseline scan ensured that the medication groups did not have pre-existing differences in alcohol cue reactivity and CBF. Topiramate has a long titration period, and consequently, patients were scanned after about 6 weeks of dosing, including a full week at maximum dosage. Although this approach allowed for a more accurate assessment of topiramate’s effects, the long interval between scans led to patient attrition.

In conclusion, this preliminary study combined functional neuroimaging with a placebo-controlled RCT of topiramate for treating AUD. Among heavy drinking, treatment-seeking individuals with AUD, topiramate reduced both alcohol cue-elicited activation of several regions of the dopaminergic mesocorticolimbic reward circuit (i.e., left VS, bilateral OFC, and medial OFC) and heavy drinking and that these changes were significantly correlated. These findings provide evidence that topiramate’s attenuation of alcohol cue-elicited brain activation is a key element of the drug’s neurobiological mechanism of action in reducing heavy drinking.

## Funding and disclosure

This study was supported by National Institutes of Health grants K23 AA023894 (Wetherill) and R01 AA023192 from the National Institute on Alcohol Abuse and Alcoholism and the VISN 4 Mental Illness Research, Education and Clinical Center, U.S. Department of Veterans Affairs. The funding sources had no role in the design of this study or in its execution, analyses, interpretation of the data, or decision to submit the results for publication. HRK is a member of an advisory board for Dicerna and the American Society of Clinical Psychopharmacology’s Alcohol Clinical Trials Initiative, which during the past 3 years was supported by AbbVie, Alkermes, Dicerna, Ethypharm, Indivior, Lilly, Lundbeck, Otsuka, Pfizer, Arbor and Amygdala Neurosciences. HRK is also named as an inventor on PCT patent application #15/878,640 entitled: “Genotype-guided dosing of opioid agonists,” filed January 24, 2014. Other authors have declared that there are no competing or conflicts of interest.

## Supplementary information

Supplemental Materials

## References

[1] World Health Organization. Global status report on alcohol and health 2018. Licence: CC BY-NC-SA 3.0 IGO. Geneva: World Health Organization; 2018.

[2] Substance Abuse and Mental Health Services Administration. 2018 National Survey on Drug Use and Health (NSDUH). Table 2. 1b–Tobacco product and alcohol use in lifetime, past year, and past month among persons aged 12 or older, by age group: percentages, 2017 and 2018. https://www.Samhsa.Gov/data/sites/default/files/cbhsq-reports/nsduhdetailedtabs2018r2/nsduhdettabssect2pe2018.Htm#tab2-1b. Accessed 11 May 2020.

[3] Sinha R (2011). New findings on biological factors predicting addiction relapse vulnerability. Curr Psychiatry Rep.

[4] Everitt BJ, Robbins TW (2005). Neural systems of reinforcement for drug addiction: from actions to habits to compulsion. Nat Neurosci.

[5] Grusser SM, Wrase J, Klein S, Hermann D, Smolka MN, Ruf M (2004). Cue-induced activation of the striatum and medial prefrontal cortex is associated with subsequent relapse in abstinent alcoholics. Psychopharmacology.

[6] Schacht JP, Anton RF, Myrick H (2013). Functional neuroimaging studies of alcohol cue reactivity: A quantitative meta-analysis and systematic review. Addict Biol.

[7] Vollstadt-Klein S, Loeber S, Kirsch M, Bach P, Richter A, Buhler M (2011). Effects of cue-exposure treatment on neural cue reactivity in alcohol dependence: a randomized trial. Biol Psychiatry.

[8] Loeber S, Croissant B, Heinz A, Mann K, Flor H (2006). Cue exposure in the treatment of alcohol dependence: effects on drinking outcome, craving and self-efficacy. Br J Clin Psychol.

[9] Vollstadt-Klein S, Wichert S, Rabinstein J, Buhler M, Klein O, Ende G (2010). Initial, habitual and compulsive alcohol use is characterized by a shift of cue processing from ventral to dorsal striatum. Addiction.

[10] Nguyen-Louie TT, Courtney KE, Squeglia LM, Bagot K, Eberson S, Migliorini R (2018). Prospective changes in neural alcohol cue reactivity in at-risk adolescents. Brain Imaging Behav.

[11] Kranzler HR, Soyka M (2018). Diagnosis and pharmacotherapy of alcohol use disorder: a review. JAMA..

[12] Johnson BA, Ait-Daoud N, Bowden CL, DiClemente CC, Roache JD, Lawson K (2003). Oral topiramate for treatment of alcohol dependence: a randomised controlled trial. Lancet.

[13] Baltieri DA, Daro FR, Ribeiro PL, de Andrade AG (2008). Comparing topiramate with naltrexone in the treatment of alcohol dependence. Addiction.

[14] Rubio G, Martinez-Gras I, Manzanares J (2009). Modulation of impulsivity by topiramate: implications for the treatment of alcohol dependence. J Clin Psychopharmacol.

[15] Blodgett JC, Del Re AC, Maisel NC, Finney JW (2014). A meta-analysis of topiramate’s effects for individuals with alcohol use disorders. Alcohol Clin Exp Res.

[16] Kranzler HR, Covault J, Feinn R, Armeli S, Tennen H, Arias AJ (2014). Topiramate treatment for heavy drinkers: moderation by a GRIK1 polymorphism. Am J Psychiatry.

[17] Kranzler HR, Morris PE, Pond T, Crist RC, Kampman KM, Hartwell EE, et al. Prospective pharmacogenetic study of topiramate for treating alcohol use disorder. Neuropsychopharmacology. 2021, in press. 10.1038/s41386-020-00945-9 PMC8209023 33568796

[18] Gibbs JW, Sombati S, DeLorenzo RJ, Coulter DA (2000). Cellular actions of topiramate: Blockade of kainate-evoked inward currents in cultured hippocampal neurons. Epilepsia.

[19] Johnson BA, Ait-Daoud N, Wang XQ, Penberthy JK, Javors MA, Seneviratne C (2013). Topiramate for the treatment of cocaine addiction: a randomized clinical trial. JAMA Psychiatry.

[20] Leggio L, Schwandt ML (2014). Topiramate for alcoholism treatment: Novel pharmacogenetic evidence for the journey to personalized medicine?. Int J Neuropsychopharmacol.

[21] Farokhnia M, Browning BD, Leggio L (2019). Prospects for pharmacotherapies to treat alcohol use disorder: an update on recent human studies. Curr Opin Psychiatry.

[22] Paparrigopoulos T, Tzavellas E, Karaiskos D, Kourlaba G, Liappas I (2011). Treatment of alcohol dependence with low-dose topiramate: an open-label controlled study. BMC Psychiatry.

[23] Rubio G, Ponce G, Jimenez-Arriero MA, Palomo T, Manzanares J, Ferre F (2004). Effects of topiramate in the treatment of alcohol dependence. Pharmacopsychiatry.

[24] Miranda R, MacKillop J, Monti PM, Rohsenow DJ, Tidey J, Gwaltney C (2008). Effects of topiramate on urge to drink and the subjective effects of alcohol: a preliminary laboratory study. Alcohol Clin Exp Res.

[25] Miranda R, MacKillop J, Treloar H, Blanchard A, Tidey JW, Swift RM (2016). Biobehavioral mechanisms of topiramate’s effects on alcohol use: an investigation pairing laboratory and ecological momentary assessments. Addict Biol.

[26] Wechsler D. Wechsler abbreviated scale of intelligence manual. San Antonio: Harcourt Brace & Company; 1999.

[27] First MB, Spitzer RL, Gibbon M, Williams JB. Structured Clinical Interview for DSM-IV-TR axis I disorders, research version, patient edition with psychotic screen (SCID-I/P w/psy screen). New York: New York Psychiatric Institute; 2001.

[28] American Psychiatric Association. DSM-5 task force: diagnostic and statistical manual of mental disorders: DSM-5™, 5th ed. Arlington, VA :American Psychiatric Publishing; 2013.

[29] Sobell L, Sobell M. Timeline follow-back: a technique for assessing self-reported alcohol consumption. In: Clifton AJ, Totowa NJ, Editors. Measuring alcohol consumption. Totowa, NJ: Humana Press; 1992. p. 41–65.

[30] Miller WR, Tonigan JS, Longabaugh R. National Institute on Alcohol Abuse and Alcoholism (U.S.): The Drinker Inventory of Consequences (DrinC): an instrument for assessing adverse consequences of alcohol abuse: Test manual, Rockville, MD: U.S. Dept. of Health and Human Services, Public Health Service, National Institutes of Health, National Institute on Alcohol Abuse and Alcoholism; 1995.

[31] Sullivan JT, Sykora K, Schneiderman J, Naranjo CA, Sellers EM (1989). Assessment of alcohol withdrawal: The revised clinical institute withdrawal assessment for alcohol scale (CIWA-aR). Br J Addict.

[32] Pettinati HM, Weiss RD, Dundon W, Miller WR, Donovan D, Ernst DB (2005). A structured approach to medical management: a psychosocial intervention to support pharmacotherapy in the treatment of alcohol dependence. J Stud Alcohol Suppl.

[33] Floyd TF, Ratcliffe SJ, Wang J, Resch B, Detre JA (2003). Precision of the CASL-perfusion MRI technique for the measurement of cerebral blood flow in whole brain and vascular territories. J Magn Reson Imaging.

[34] Wilson SJ, Sayette MA, Fiez JA, Brough E (2007). Carry-over effects of smoking cue exposure on working memory performance. Nicotine Tob Res.

[35] Waters AJ, Sayette MA, Franken IH, Schwartz JE (2005). Generalizability of carry-over effects in the emotional stroop task. Behav Res Ther.

[36] Wang Z, Aguirre GK, Rao H, Wang J, Fernandez-Seara MA, Childress AR (2008). Empirical optimization of ASL data analysis using an ASL data processing toolbox: Asltbx. Magn Reson Imaging.

[37] Greve DN, Fischl B (2009). Accurate and robust brain image alignment using boundary-based registration. Neuroimage.

[38] Alsop DC, Detre JA, Golay X, Gunther M, Hendrikse J, Hernandez-Garcia L (2015). Recommended implementation of arterial spin-labeled perfusion MRI for clinical applications: a consensus of the ISMRM perfusion study group and the European consortium for ASL in dementia. Magn Reson Med.

[39] Dolui S, Wolf R, Ali Nabavizadeh S, Wolk DA, Detre J. Automated quality evaluation index for 2d ASL cbf maps. International Society for Magnetic Resonance in Medicine - ISMRM; 2017;682.

[40] Schacht JP, Anton RF, Randall PK, Li X, Henderson S, Myrick H (2014). Varenicline effects on drinking, craving and neural reward processing among non-treatment-seeking alcohol-dependent individuals. Psychopharmacology.

[41] Schacht JP, Anton RF, Randall PK, Li X, Henderson S, Myrick H (2011). Stability of fMRI striatal response to alcohol cues: a hierarchical linear modeling approach. Neuroimage.

[42] Tsai HC, Zhang F, Adamantidis A, Stuber GD, Bonci A, de Lecea L (2009). Phasic firing in dopaminergic neurons is sufficient for behavioral conditioning. Science.

[43] Schultz W, Dayan P, Montague PR (1997). A neural substrate of prediction and reward. Science.

[44] Schultz W (2007). Behavioral dopamine signals. Trends Neurosci.

[45] Ray JP, Price JL (1993). The organization of projections from the mediodorsal nucleus of the thalamus to orbital and medial prefrontal cortex in macaque monkeys. J Comp Neurol.

[46] Volkow ND, Fowler JS (2000). Addiction, a disease of compulsion and drive: Involvement of the orbitofrontal cortex. Cereb Cortex.

[47] Keiflin R, Reese RM, Woods CA, Janak PH (2013). The orbitofrontal cortex as part of a hierarchical neural system mediating choice between two good options. J Neurosci.

[48] Namboodiri VMK, Otis JM, van Heeswijk K, Voets ES, Alghorazi RA, Rodriguez-Romaguera J (2019). Single-cell activity tracking reveals that orbitofrontal neurons acquire and maintain a long-term memory to guide behavioral adaptation. Nat Neurosci.

[49] Breslow RA, Graubard BI (2008). Prospective study of alcohol consumption in the united states: Quantity, frequency, and cause-specific mortality. Alcohol Clin Exp Res.

[50] Dawson DA, Li TK, Grant BF (2008). A prospective study of risk drinking: at risk for what?. Drug Alcohol Depend.

[51] Jackson KM (2008). Heavy episodic drinking: determining the predictive utility of five or more drinks. Psychol Addict Behav.

