# Supplementary material for: Effects of topiramate on neural responses to alcohol cues in treatment-seeking individuals with alcohol use disorder: preliminary findings from a randomized, placebo-controlled trial
Source: Neuropsychopharmacology. 2021 Feb 8;46(8):1414–20. doi: 10.1038/s41386-021-00968-w (PMC8208990; doi:10.1038/s41386-021-00968-w)
Supplement: Supplementary file 1 — Supplemental Materials [file 41386_2021_968_MOESM1_ESM.docx]

**Supplemental Background:** The current sub-study capitalizes on the neuroimaging technique of pseudo-continuous arterial spin-labeled (pCASL) perfusion fMRI. Perfusion fMRI is a noninvasive, non-radioactive technique that is used to measure neurophysiological activity. It uses a magnetic field to induce a radio-frequency signal to reflect the brain anatomy and the changes in cerebral blood flow (CBF) in near real-time. Changes in CBF result in changes in tissue contrast measured reliably with MRI scanners. Perfusion refers to the delivery of oxygen and nutrients to tissue by means of blood flow and is regionally coupled to brain metabolism. Perfusion fMRI is distinguished from BOLD contrast, which is acquired via oxygenated vs. deoxygenated hemoglobin. Perfusion fMRI is quantitative [[1](#_ENREF_1)] and stable across time [[2](#_ENREF_2)], which facilitates the measurement of brain responses at various time points, both in response to cognitive and emotional tasks, such as cue exposure [[3-5](#_ENREF_3)] and also in the brain in the resting condition (without provocation) [[6](#_ENREF_6), [7](#_ENREF_7)]. As such, it is ideal for longitudinal studies examining brain modifications induced by pharmacological agents. Another particularly important feature is that noise characteristics are stable over the entire frequency spectrum, making it suitable for studying low-frequency events in brain function such as craving and other emotional states that accrue over time [[1](#_ENREF_1), [8](#_ENREF_8)]. Consequently, in our paradigm, this stable and quantitative technique has distinct advantages over BOLD imaging for revealing the neurobiological mechanisms underlying task performance (i.e., cue exposure) and its modulation by pharmacological agents.

**Supplemental Methods:** The current sub-study started recruitment nine months after the main study was initiated, which reduced the number of potential patients to 149. For six months of the 3.5 years of overlap between the main and sub-study, the MRI scanner was not functioning due to a catastrophic event (fire), which further reduced the number of potential subjects to 101. Of this number, 31 were not screened/consented for the sub-study due to age or BMI exclusions. An additional 32 main study patients declined participation in the sub-study and thus were not screened, consented, or enrolled. Thus, 38 main study patients were consented, screened, and enrolled in the current study.

\

**Supplemental Figure 1: CONSORT Diagram**


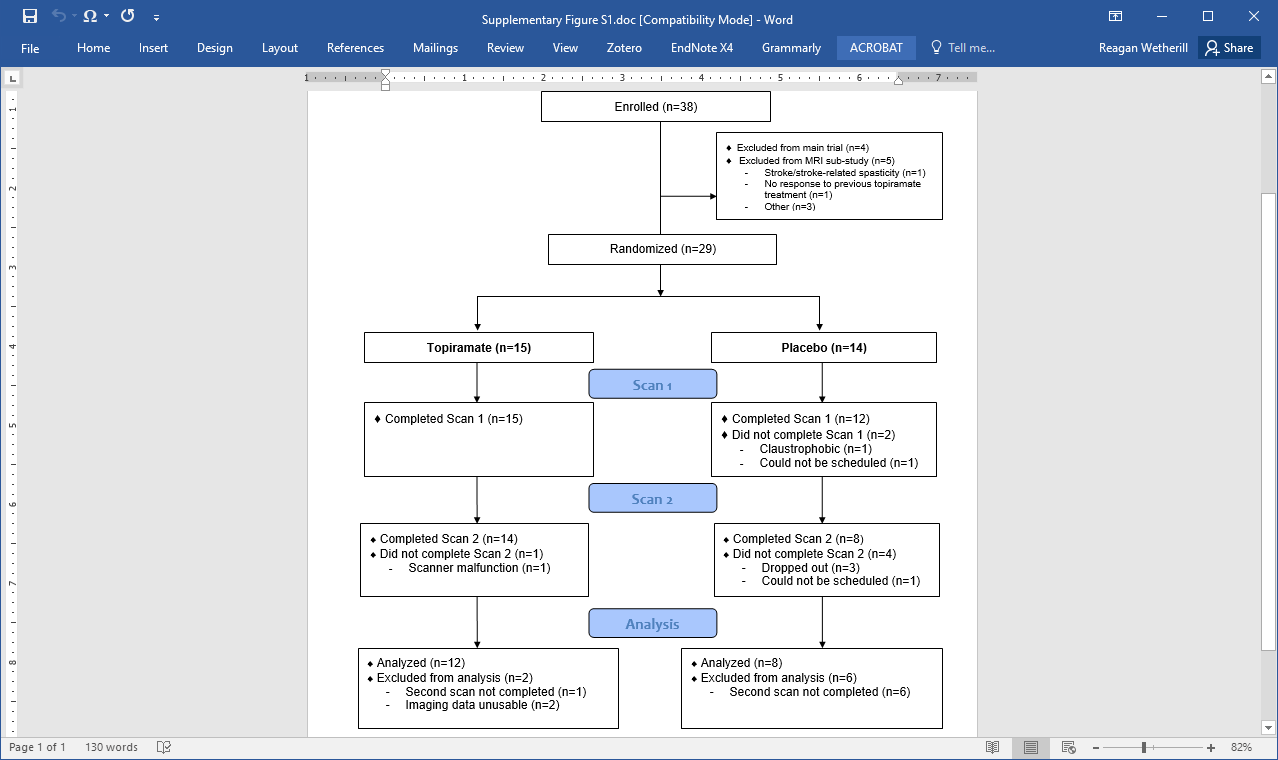


**References**

1. Alsop DC, Detre JA. Reduced transit-time sensitivity in noninvasive magnetic resonance imaging of human cerebral blood flow*.* Journal of Cerebral Blood Flow and Metabolism. 1996;16:1236-1249.

2. Hermes M, Hagemann D, Britz P, Lieser S, Rock J, Naumann E*, et al.* Reproducibility of continuous arterial spin labeling perfusion mri after 7 weeks*.* MAGMA. 2007;20:103-115.

3. Franklin TR, Jagannathan K, Wetherill RR, Johnson B, Kelly S, Langguth J*, et al.* Influence of menstrual cycle phase on neural and craving responses to appetitive smoking cues in naturally cycling females*.* Nicotine Tob Res. 2015;17:390-397.

4. Wetherill RR, Young KA, Jagannathan K, Shin J, O'Brien CP, Childress AR*, et al.* The impact of sex on brain responses to smoking cues: A perfusion fMRI study*.* Biology of Sex Differences. 2013;4:9.

5. Wetherill RR, Jagannathan K, Lohoff FW, Ehrman R, O'Brien CP, Childress AR*, et al.* Neural correlates of attentional bias for smoking cues: Modulation by variance in the dopamine transporter gene*.* Addict Biol. 2014;19:294-304.

6. Wang K, Liang M, Wang L, Tian L, Zhang X, Li K*, et al.* Altered functional connectivity in early alzheimer's disease: A resting-state fMRI study*.* Hum Brain Mapp. 2007;28:967-978.

7. Scheef L, Manka C, Daamen M, Kuhn KU, Maier W, Schild HH*, et al.* Resting-state perfusion in nonmedicated schizophrenic patients: A continuous arterial spin-labeling 3.0-T MR study*.* Radiology. 2010;256:253-260.

8. Detre JA, Leigh JS, Williams DS, Koretsky AP. Perfusion imaging*.* Magn Reson Med. 1992;23:37-45.
